# Targeting MUC1 with fisetin in oral squamous cell carcinoma

**DOI:** 10.1016/j.gendis.2024.101357

**Published:** 2024-06-20

**Authors:** Qian Wang, Hongyan Zhang, Shuzhen Xiang, Lan Zhang, Jiajia Fan, Zengyan Xu, Fengfei Zhao, Minda Liu, Yanshu Li, Wei Dai

**Affiliations:** a Department of Oral Maxillofacial-Head and Neck Surgery, School of Stomatology, China Medical University, Oral Diseases Laboratory of Liaoning, Shenyang, Liaoning 110000, China; b Department of Cell Biology, Key Laboratory of Cell Biology, Ministry of Public Health, China Medical University, Shenyang, Liaoning 110122, China; c Key Laboratory of Medical Cell Biology, Ministry of Education, China Medical University, Shenyang, Liaoning 110122, China

Poor prognosis is associated with oral squamous cell carcinoma (OSCC), an aggressive form of malignant tumor.^1^ Developing effective targeted therapies against OSCC is anticipated to have significant clinical implications. Fisetin (3, 3′, 4′, 7-tetrahydroxyflavone), a natural flavonoid, the most common phytochemical found in a variety of fruits and vegetables, may bring several therapeutic potential benefits to people.^2^ Investigating the pharmacological impact of natural flavonoid fisetin on the management of OSCC was the aim of the current investigation. By focusing on the tumor-associated antigen MUC1 (mucin 1), fisetin prevents OSCC cells from transforming malignant. This study aims to provide new diagnostic indicators and therapeutic targets for the diagnosis and treatment of OSCC by exploring the role and mechanism of fisetin in OSCC.

Fisetin, formerly referred to as a novel anti-cancer properties in OSCC cancer cells, is a natural flavonoid compound (Fig. 1A). To investigate whether fisetin retained the anti-tumor efficacy, we design a series of research strategies to detect biological characteristics of fisetin (Fig. S1A). Our data showed that fisetin significantly inhibited the cell growth (Fig. S1B) and colony formation (Fig. 1B) ability of OSCC cells. Furthermore, a scratch assay was conducted to detect the migration abilities, which revealed that migrated cells were significantly suppressed after fisetin treatment (Fig. 1C). Moreover, OSCC cells with fisetin treatment were more susceptible to chemotherapy-induced cell apoptosis by flow cytometry-PI/FITC double staining (Fig. 1D). Thus, these results supported that fisetin inhibits OSCC cell proliferation and metastasis and promotes OSCC cell apoptosis.

**Figure 1:**
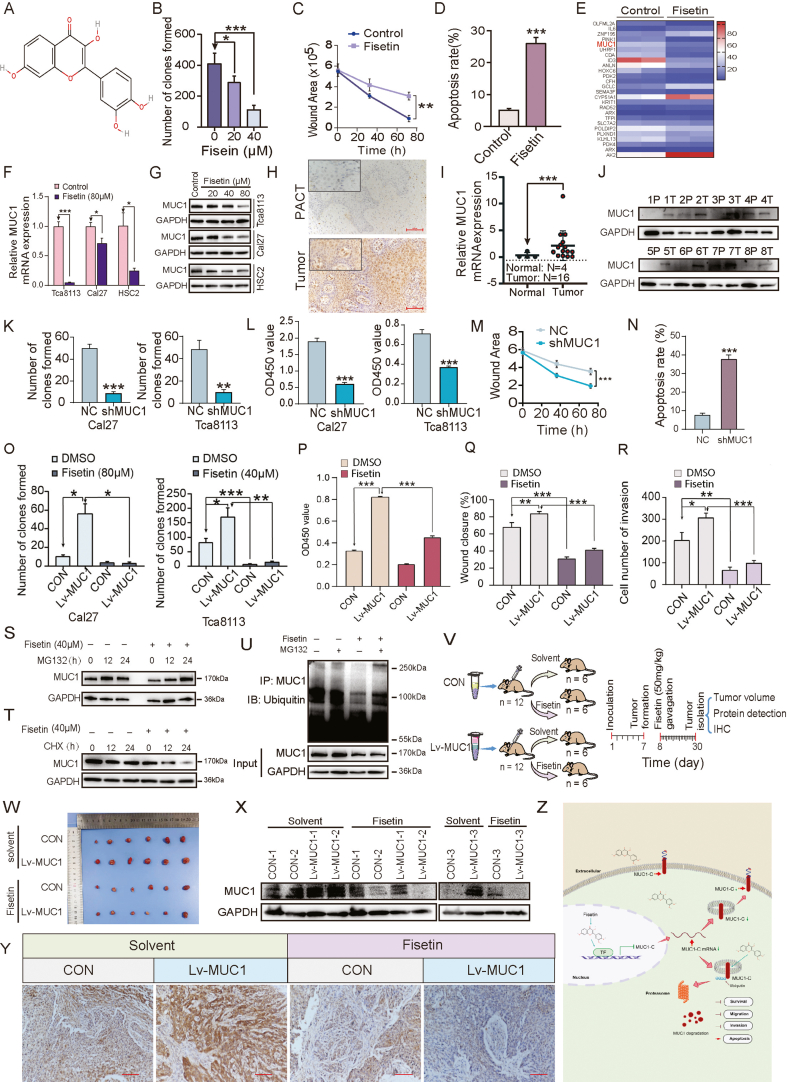
The biological effects and mechanisms of fisetin for OSCC therapy by inhibition of MUC1. **(A)** Chemical structure of fisetin. **(B)** Colony-formation assay of HSC4 cells treated with different concentrations of fisetin. **(C)** The scratch assay detected the healing at 32 h and 72 h. **(D)** Cell-apoptosis analysis of the percentage of apoptotic cells in Cal27 cells treated with 40 μM fisetin for 24 h. **(E)** The heat map represents the significantly altered gene expression levels in Tca8113 cells treated with fisetin for 24 h. **(F, G)** Reverse transcription-quantitative PCR and immunoblotting verification of MUC1 in the Tca8113, Cal27, and HSC2 cells after treatment with fisetin for 24 h. **(H)** Representative immunohistochemistry (IHC) images of MUC1 in para-cancerous tissues (PACT; *n* = 10) and in primary tumors (tumor; *n* = 83). Scale bar, 100 μm (left). MUC1 expression is shown by the pathologic score of cells expressing MUC1 positivity at any intensity (right). **(I)** Comparison of MUC1 gene expression between normal and OSCC tumor tissues in the GSE31056 dataset. **(J)** MUC1 was detected by western blot in eight pairs of matched para-cancerous tissues and OSCC tissues. P, PACT; T, tumor. **(K)** A colony photograph and the number of colonies formed of Cal 27 and Tca8113 cells after being infected with NC or shMUC1. **(L)** Cell viability of OSCC cells was measured by CCK-8 assay. **(M)** The scratch assay was used to detect the healing at 36 h and 72 h. **(N)** Apoptosis after MUC1 silencing in Cal27 cells was assessed using annexin V and PI double staining-based FACS analysis. **(O)** Colony formation assay for Cal27 and Tca8113 cells treated with DMSO or fisetin for 14 days stably after being infected with lentivirus CON or Lv-MUC1. **(P)** Cell viability of Tca8113 and Cal27 cell lines was measured by CCK-8 assay. **(Q)** The scratch assay was used to detect the healing at 24 h in HSC4 cells. **(R)** The invasion ability was determined after Con or Lv-MUC1 in HSC4 cells was treated with or without fisetin (40 μM) using the transwell assay. **(S)** Western blot analysis for MUC1 in Cal27 cells treated with DMSO or 40 μM fisetin in the presence of 20 μM MG132 at the indicated time point. **(T)** Western blot analysis for MUC1 in Cal27 cells treated with DMSO or 40 μM fisetin in the presence of 50 μg/mL cycloheximide (CHX) at the indicated time point. **(U)** Ubiquitination assays for MUC1 treatment with or without fisetin. **(V)** A diagram of the time of tumor formation in BALB/c nude mice. **(W)** The growth curves of tumor volumes were measured in xenograft mice of each group every 2–3 days (*n* = 6). Two-way ANOVA test. **(X)** Proteins from mouse tumor tissues were extracted for western blot detection and expression analysis. **(Y)** The IHC appearance of MUC1 (brown) in sections of tumor xenografts and representative images of IHC staining (left) and quantification (right) of MUC1 signals by percentages of brown color staining intensity in IHC images. Scale bar, 100 mm. **(Z)** MUC1 was regulated by fisetin. Proposed model of fisetin regulation of MUC1 gene and protein expression. OSCC, oral squamous cell carcinoma; MUC1, mucin 1.

To further excavate the molecular effects elicited by fisetin, we analyzed the transcriptomic data after treatment with DMSO or fisetin by the nanopore third-generation whole genome sequencing (Fig. 1E). MUC1, a heterodimeric transmembrane glycoprotein,^3^ was significantly down-regulated in the fisetin treatment cell lines by quantitative PCR assays (Fig. 1F). Then, the protein expression of MUC1 was decreased by fisetin treatment in a dose-dependent manner as indicated in OSCC cells (Fig. 1G). Therefore, these findings suggested that MUC1 was down-regulated by fisetin in OSCC cells, and MUC1 could be a potential therapy target for fisetin in OSCC clinical treatment.

To investigate the expression and biodistribution of MUC1 in OSCC, we performed immunohistochemical staining for MUC1 on the OSCC tissues (tumor) and the para-cancerous tissues from the Hospital of Stomatology of China Medical University, and we found that MUC1 was localized in both the cytoplasm and nucleus (Fig. 1H). In the analysis of 83 cases, the protein expression levels of MUC1 were significantly elevated in OSCC tissues compared with para-cancerous tissues. Then, the GEO database illustrated that the expression levels of MUC1 were higher in 16 patients with OSCC tissues than in normal tissues from four individuals (Fig. 1I). Consistent with these, our western blot results showed that MUC1 was up-regulated in OSCC tumor tissues (Fig. 1J). To determine the gene levels of MUC1 in OSCC tissues, we analyzed gene expression of MUC1 from eight OSCC patients. MUC1 gene was detected at a higher level in OSCC tissues compared with the para-cancerous tissues (Fig. S2A). To further confirm the expression of MUC1 in OSCC, western blot assays confirmed that MUC1 was also overexpressed in Tca8113, Cal27, HSC2, and SCC9 cell lines, while its low expression was found in HSC4 cell line (Fig. S2B). These data suggest that MUC1 acts as an oncoprotein and serves as a novel therapeutic target for OSCC.

Considering that fisetin acts as an inhibitor of MUC1 proteins, we next studied the effect of MUC1 down-regulation in OSCC progression. We first generated targeted MUC1 knockout OSCC cell lines (Fig. S3A, B). Our data revealed that knockdown of MUC1 inhibited colony formation (Fig. 1K; Fig. S3C) and growth (Fig. 1L) in both Cal27 and Tca8113 cells. Moreover, MUC1 knockdown significantly inhibited migration (Fig. 1M; Fig. S3D) and increased apoptosis in Cal27 cells (Fig. 1N; Fig. S3E). Overall, our results reveal that MUC1 plays an important role in promoting OSCC progression.

Fisetin reverses MUC1-induced OSCC cell malignant transformation. To elucidate the effect of fisetin, we elevated MUC1 expression by stably infecting with MUC1 lentivirus. Quantitative PCR and western blot showed that MUC1 was significantly increased in the OSCC cells transfected with Lv-MUC1 (Fig. S4A, B). Fisetin at micromolar concentration significantly reduced MUC1-induced colony formation (Fig. 1O; Fig. S4C) and proliferation (Fig. 1P). The results showed that inhibition of MUC1 could be effective in reducing or blocking cancer growth by fisetin treatment. Furthermore, scratch wound and transwell assays showed that fisetin had a significant effect on migration (Fig. 1Q; Fig. S4D) and invasion (Fig. 1R; Fig. S4E) of HSC4 cells. These results indicated the role of fisetin in MUC1-induced OSCC cell proliferation, colony formation, migration, and invasion.

Fisetin regulates MUC1 stability in OSCC cells. We examined how fisetin affected MUC1 expression. Upon treatment with the proteasome inhibitor MG132,^4^ MUC1 expression significantly accumulated in the presence of fisetin (Fig. 1S), thereby suggesting that the ubiquitin-proteasome pathway may be required for fisetin-mediated reduction of MUC1 protein abundance. Indeed, the expression of MUC1 was dramatically decreased in Cal27 cells treated with the protein synthesis inhibitor cycloheximide (Fig. 1T). Consistent with these findings, the ubiquitination assay revealed that fisetin significantly increased the ubiquitin levels of endogenous MUC1 protein in OSCC cells (Fig. 1U). The findings imply that the fisetin treatment causes significant instability in the MUC1 protein.

Fisetin impairs MUC1-induced cell proliferation in OSCC xenografts. Our results suggest that MUC1 targeting may be a promising therapeutic strategy in patients with OSCC. Therefore, we further investigated whether fisetin could suppress tumor growth in xenograft models. To determine the pharmacological activity, we divided nude mice into four groups. First, we generated an orthotopic OSCC xenograft model by subcutaneous injection of Cal27 cells with infection CON or Lv-MUC1 lentivirus into nude mice. Then, we treated the tumor-bearing mice with vehicle (corn oil) alone or fisetin seven days later (Fig. 1V). The volumes of subcutaneous xenografts were dramatically increased in ectopic MUC1 expression mice compared with control mice, whereas the administration of fisetin obviously suppressed MUC1-induced tumor formation in the murine models of OSCC (Fig. 1W). Finally, western blot assay and immunohistochemistry data of MUC1 expression and staining in the tissues of xenografts showed that the administration of fisetin dramatically decreased the levels of MUC1 protein (Fig. 1X, Y). These results indicated that fisetin suppressed tumor growth in OSCC xenograft models by targeting the MUC1 protein, suggesting fisetin to be a promising therapeutic candidate for OSCC (Fig. 1Z).

In summary, we found that fisetin efficiently inhibited MUC1 tumorigenesis, implying that it is an MUC1-specific inhibitor. Although these results add to the scientific understanding of the anti-cancer mechanism of fisetin in OSCC therapies, more clinical trials need to be performed to explore the anti-cancer potential and mechanism of action of fisetin and its optimum therapeutic dose. Overall, our findings prove for the first time, that fisetin, an MUC1 inhibitor, is a highly effective treatment for OSCC cancer.

## Ethics declaration

All the samples were obtained from the Hospital of Stomatology of China Medical University. All animal experiments were approved by the Animal Care Committee of China Medical College (approval number: KT2023074).

## Author contributions

Y.L. and Q.W. conceived the study and W.D. and M.L. carried out its design. H.Z., Z.X., S.X., J.F., F.Z., and Q.W. performed the experiments. Q.W. collected clinical samples. Y.L. and Q.W. analyzed the data and wrote the manuscript. H.Z. revised the manuscript. All the authors read and approved the final manuscript.

## Funding

This work was supported by the 10.13039/501100001809National Natural Science Foundation of China (No. 81902701 to Y.L., 82103649 to H.Z.), the 10.13039/501100005047Natural Science Foundation of Liaoning Province, China (No. 20180530037 to Y.L., 2022-MS-183 to W.D.).

## Data availability

The authors confirm that the data supporting the findings of this study is available within the article and its supplementary materials.

## Conflict of interests

The authors declared no conflict of interests.
