# Supplementary material for: Targeting MUC1 with fisetin in oral squamous cell carcinoma
Source: Genes Dis. 2024 Jun 20;12(3):101357. doi: 10.1016/j.gendis.2024.101357 (PMC11804558; doi:10.1016/j.gendis.2024.101357)
Supplement: Multimedia component 1 [file mmc1.docx]

**Supplementary Material and Methods**

**Cell culture and reagents**

Human OSCC cell lines Tca8113 and Cal27 were purchased from Shanghai Genechem, and HSC2, HSC4, and SCC9 were purchased from Jiangsu KeyGEN BioTECH. The cells were thawed according to the manufacturer's protocol and grown in DMEM medium (HyClone, USA) containing 10% fetal bovine serum (FBS, Biological Industries (BI), China), high glucose, and 1% ampicillin-streptomycin solution at 37 °C in a humidified atmosphere of 95% air and 5% CO_2_. Fisetin were purchased from Sigma Aldrich (St. Louis, MO, USA, PHL82542), MedChemExpress (MCE, China, HY-N0182). Cycloheximide (CHX) and MG132 were purchased from MedChemExpress (MCE, China, HY-12320 and HY-13259).

**Clinical specimens and ethics statement**

8 paired (fresh-frozen) OSCC tissue samples and matched adjacent non-tumor tissue samples were collected for the detection of gene and protein expression from the same patient. A total of 100 formalin-fixed paraffin-embedded tissues and ten adjacent non-tumor tissues were collected for immunohistochemistry (IHC) staining. All the samples were obtained from Hospital of Stomatology of China Medical University. The study protocol was approved by the Hospital of Stomatology of China Medical University Ethics Committee. All experiments were performed in compliance with the relevant regulations, and all patients provided written informed consent.

**IHC staining**

IHC assay was performed as previously described^18^. The tumor tissues were embedded in paraffin and cut into 4-μm sections for IHC staining. After the slides were cut, xylene and ethanol were used for dewaxing and dehydration. Next, the slides were processed for antigen retrieval. Then 3% H_2_O_2_ was dropped onto the slides for 8 min. The tissues were incubated with primary antibody MUC1 at 4°C overnight. The slides were incubated with HRP-IgG secondary antibody for 20 min at 37°C. Finally, the slides were stained with diaminobenzidine (DAB) for 2 min and restained with hematoxylin. The IHC score (IS) was calculated based on the staining intensity and percentage of positively stained cells.

**Lentivirus generation of stable clones**

The lentivirus carrying MUC1 full length gene or shRNA-MUC1 was constructed by GeneChem Co. LTD (Shanghai, China). For stable transfection of RNA interference (RNAi), predesigned RNAi-expressing lentivirus particles were synthesized (GeneChem, Shanghai, China) using the target sequence 5′-GGGATACCTACCATCCTAT-3’. Lentiviral infection of OSCC cells was performed according to the manufacturer’s protocols. Briefly, cells were infected with the lentivirus for 48 h and selected with 2μg/ml of puromycin. The surviving cells were cultured for further research.

**Cell viability**

Cell viability was evaluated by the Cell Counting Kit-8 (CCK-8, Beyotime, Shanghai, China) assay following the manufacturer’s instructions. Cells in the logarithmic growth phase were seeded into 96-well culture plates and treated with Fisetin for the indicated concentrations and time-points. The medium was replaced with fresh basal medium containing 10% (v/v) CCK-8 reagent for 2 h at 37 °C. The optical density (O.D.) was measured at 450 nm with a microplate reader.

**Colony formation assay**

Colony formation assay of OSCC cells was carried out by plating stable knockdown or overexpression of MUC1 cells at a density of 200 cells/well in a 6-well plate and then different concentrations of Fisetin were added. After 7-14 days of incubation, cells in each group were washed three times with phosphate buffered saline (PBS), fixed with methanol for 10 min and stained with 1% crystal violet. The number of colonies were counted for data analysis.

**Wound healing assay**

Wound healing assay was performed to determine the migratory ability of the cells. Infected cells were incubated in 12-well plates and grown to confluence. The cell monolayer was scraped with a 200 μl micro-pipette tip, and the cell debris were washed three times with PBS. Then the medium was changed to DMEM medium without FBS. The wound areas were micrographed at indicated time points. The wound healing rate (%) was evaluated by comparing differences in wound width or area and all assays were performed in triplicate.

**Invasion and migration assays**

Transwell invasion and migration assays were performed according to the manufacturer’s protocol. Cells were seeded into the upper chamber with serum-free medium and incubated at 37 ℃. The cells adhering to the lower surface were fixed with 10% methanol for 10 min and stained with 0.1 % Crystal Violet for 10 min. Then, the cells on the upper surface of the filters were gently wiped with a cotton swab. Migration assay was conducted similarly to the invasion assay except for the Matrigel coating. The results were analyzed by Graph Pad software.

**RNA extraction and quantitative real-time PCR**

TRIzol reagent (Invitrogen, Carlsbad, CA, USA) was used to extract total RNA from treated cells according to the manufacturer’s instructions. cDNA was prepared and obtained using the PrimeScript RT Master Mix Perfect Real Time Kit (TaKaRa, Otsu, Shiga, Japan). Quantitative real-time PCR (qPCR) was performed in a total volume of 20 μ by using TB Green Master (TaKaRa, Japan) on a 7500 Real-Time PCR System (Applied Biosystems, Foster City, CA, USA). Relative amounts of complementary DNA were calculated using the Ct method and normalized to GAPDH. The specific primer sequences for qPCR were as follows: MUC1 forward: 5’-TGCTTACAGCTACCACAGCC-3’ and reverse: 5’- GCTGGGCACTGAACTTCTCT-3’; GAPDH forward: 5’-AGATAAGACCATCATCAT-3’ and reverse: 5’-AGATAACCACATCACTAA-3.

**Western blot analysis**

Cells were harvested and homogenized with RIPA buffer supplemented with complete protease inhibitor cocktail (Roche Diagnostics, Basel, Switzerland) on ice. After centrifugation at 12,000 ×g for 15 min at 4 °C, the total protein content of the supernatant was quantified using a Bicinchoninic Acid (BCA) Protein Assay (TaKaRa, Japan) and the supernatant was stored at -80 °C until use. Protein samples were separated by electrophoresis on 10% SDS-PAGE gels and transferred to polyvinylidene difluoride membranes (Millipore, Billerica, MA, USA). After blocking with 5% non-fatmilk dissolved in TBST for 2 h at room temperature, the membranes were incubated with the specific primary antibodies overnight at 4 °C. The blots were then incubated with horseradish peroxidase-conjugated anti-rabbit or anti-mouse immunoglobulin (IgG) secondary antibodies. The bands were visualized by chemiluminescence (Tanon, Shanghai, China). The following antibodies were used:  antibodies to MUC1 (1:2000, abcam, ab109185) or (1:1000, Affinity Biosciences, AF8524), antibodies to ubiquitin (1:2000, Cell Signaling Technology, #3933), and GAPDH (1:10,000; Shanghai Kangchen, KC-5G4). GAPDH were used as equal loading controls. The ImageJ software was used for densitometric analyses of Western blots, and the quantification results were normalized to the loading control.

**Coimmunoprecipitation analysis**

Cells were washed with ice-cold PBS and lysed in NP-40 lysis buffer containing PMSF and protease inhibitor on ice. The cell lysates were centrifuged at 12,000 ×g for 10 min at 4 °C. After preclearing the lysates with Protein A Sepharose™ CL-4B (Cytiva, 17078001) for 30 min at 4 °C, the supernatants were incubated with the indicated antibodies overnight at 4 °C to enrich the antigen-antibody complex. After mixing the lysate with Protein A beads for 2 h at 4 °C, the immunocomplex was washed with PBS thrice. Finally, appropriate SDS loading buffer was applied to dissolve the mixtures and subjected to western blot analysis.

***In vivo* tumor xenograft models**

All animal experiments were approved by the Animal Care Committee of China Medical College (approval number: KT2023074), and performed according to NIH Guidelines for the Care and Use of Laboratory Animals. In vivo subcutaneous xenograft experiments were performed in randomized three-week-old female BALB/c nude mice (Beijing Vital River Laboratory Animal Technology Co., Ltd., Beijing, China), with injection of 1 × 10^6^ cancer cells. For *in vivo* Fisetin therapeutic studies, 7 days after the inoculation, female BALB/c nude mice were blindly randomized gavaged with Fisetin (50 mg/kg) or Corn oil as control, once every day till to the 30th day for test *in vivo*. Mice were recorded for tumor volume.

**Statistical analysis**

The associations between MUC1 expression and clinicopathological parameters were analyzed by chi-square test or Fisher’s exact test. Statistical comparisons between two groups were examined using student's *t*-tests. Statistical comparisons among more than two groups were evaluated using one-way ANOVA. Survival curves were determined by the Kaplan-Meier way. All data are expressed as mean ± standard deviation (SD) from three independent experiments. SPSS software package version 14.0 (SPSS, Chicago, IL, USA) was used for data analysis. Statistical significance was described as follows: ns, not significant; **p* < 0.05; ***p* ≤ 0.01; ****p* ≤ 0.001.
