# Supplementary material for: Targeting MUC1 with fisetin in oral squamous cell carcinoma
Source: Genes Dis. 2024 Jun 20;12(3):101357. doi: 10.1016/j.gendis.2024.101357 (PMC11804558; doi:10.1016/j.gendis.2024.101357)
Supplement: Multimedia component 2 [file mmc2.docx]

**Supplementary figures and tables**


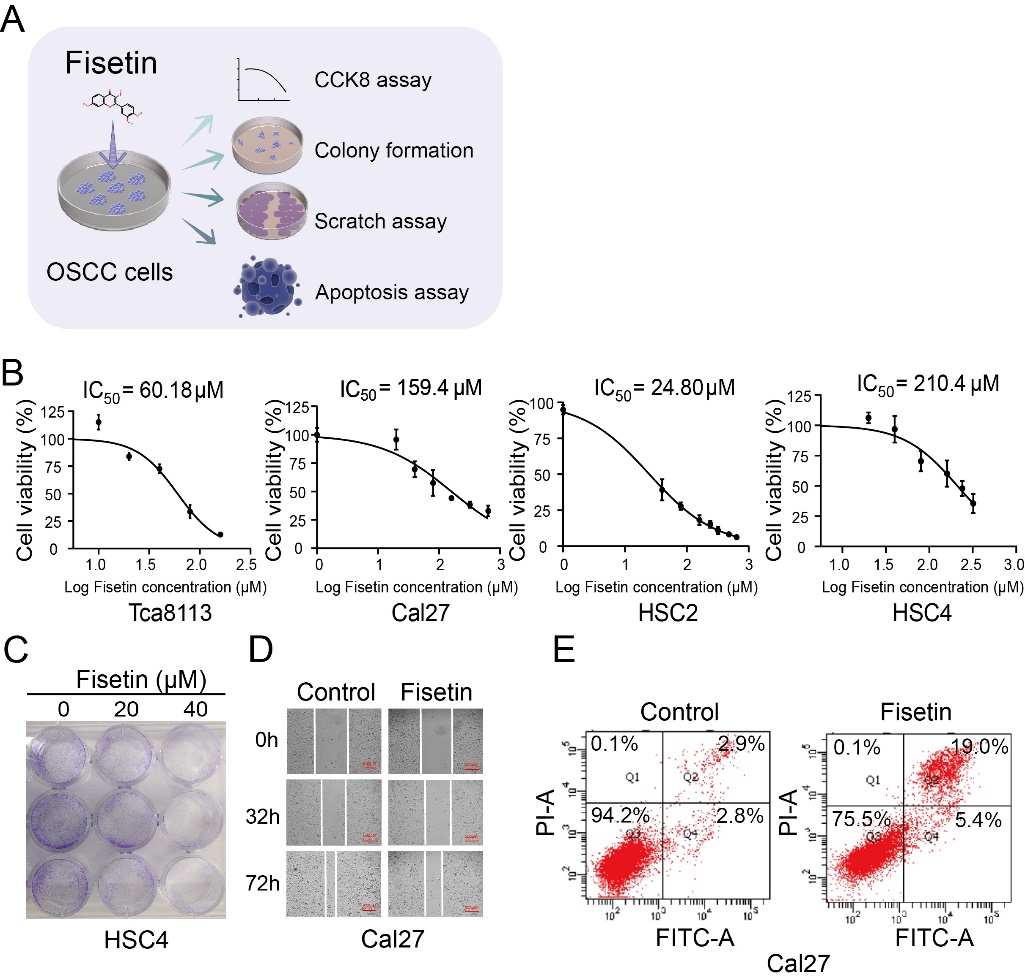


**Figure S1.** Fisetin displays anti-OSCC Activity *in vitro.* **(A)** Research strategy of the compound fisetin on the OSCC cancer cells. **(B)** The IC_50_ values of fisetin was measured by CCK-8 assay in OSCC cells. **(C)** Colony formation of HSC4 cells was detected by crystal violet staining. **(D)** The scratch assay was used to detect the healing of 32 hours and 72 hours, respectively. **(E)** Representative pictures of flow cytometry cell-apoptosis analysis and quantification of percentage of apoptotic cells in Cal27 cells treated with 40 μM Fisetin for 24 h.


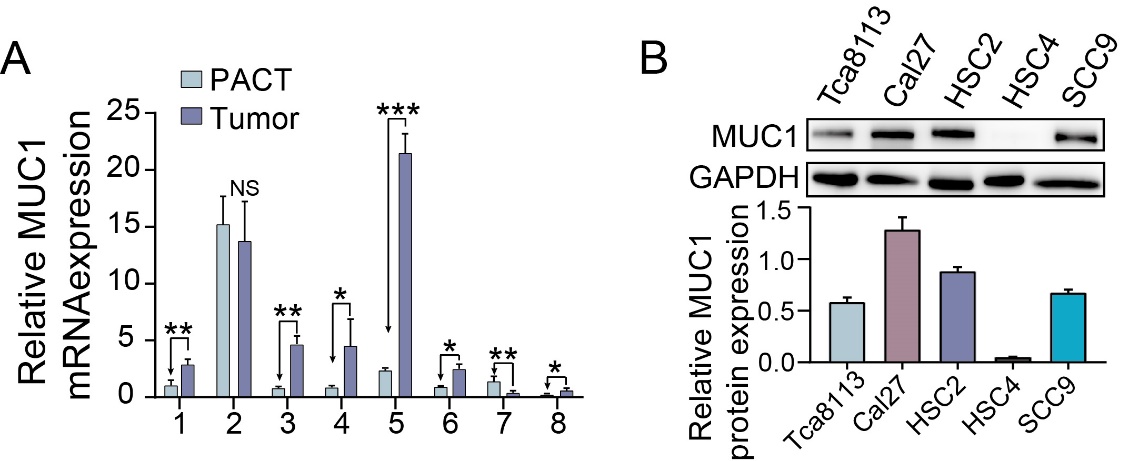


**Figure S2.** MUC1 expression is elevated in human OSCC tissues and cell lines. **(A)** Quantitative RT-PCR analysis of MUC1 mRNA level in OSCC tissues and matched PACT from 8 subjects. Data were analyzed using Student’s t-test. **(B)** Immunoblots showing protein levels of MUC1 and GAPDH in different types of OSCC cell lines.


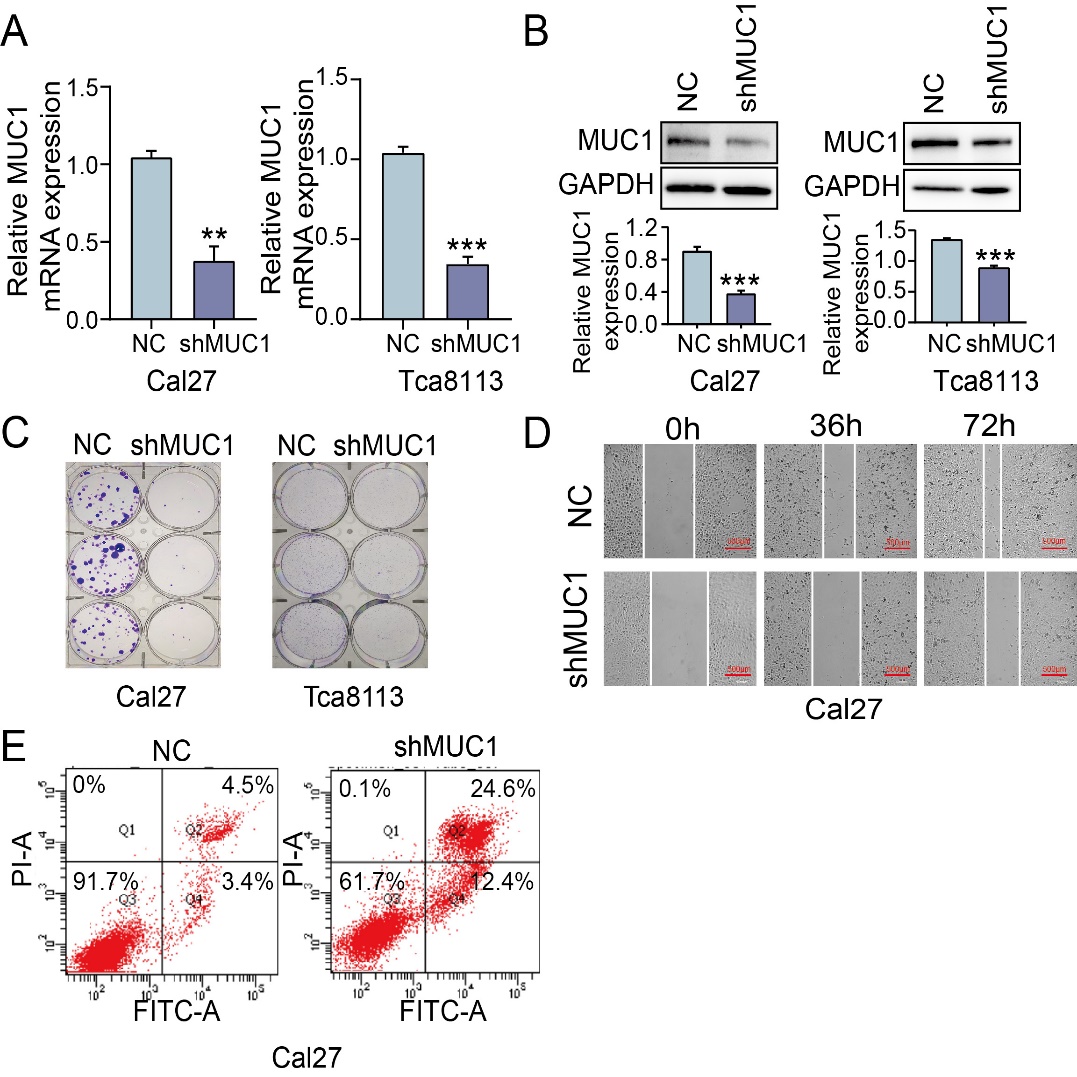


**Figure S3.** MUC1 depletion impairs OSCC progression. **(A)** RT-qPCR verification of MUC1 gene expression in Cal27 cells and Tca8113 cells infected with either control (NC) or lentivirus carrying shMUC1. **(B)** Western blotting analyses of MUC1 protein levels in Cal27 and Tca8113 cells infected with NC or shMUC1. GAPDH served as a loading control. Quantitative data of optical band densitometry are shown. **(C)** A colony photograph and the number of colonies formed of Cal 27 and Tca8113 cells after being infected with NC or shMUC1. **(D)** The scratch assay was used to detect the healing of 36 hours and 72 hours, respectively, and the healing rate of Cal27 cells in the experimental group was slowed. **(E)** Apoptosis after MUC1 silencing in Cal27 cells was assessed using Annexin V and PI double staining based FACS analysis. Q2, Q4 in the figure represent late apoptotic and early apoptotic cells, respectively.


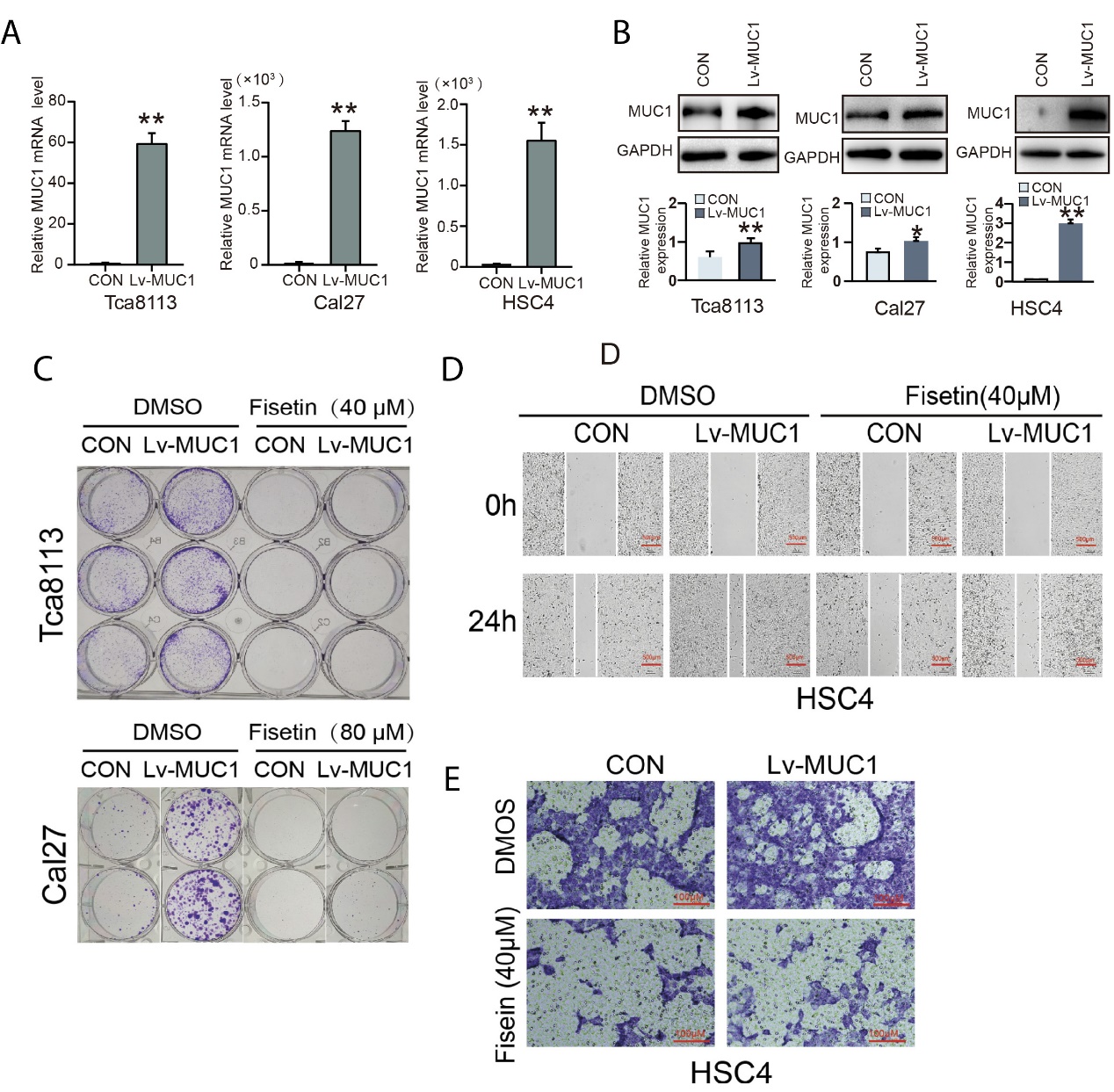


**Figure S4.** Fisetin inhibits MUC1-mediated cell proliferation, migration, and invasion. **(A)** RT-qPCR verification of MUC1 gene expression in Tca8113, Cal27, and HSC4 cells infected with lentivirus carrying CON or MUC1 genes (Lv-MUC1). **(B)** Immunoblot showing MUC1 protein levels in CON and Lv-MUC1 infected Tca8113, Cal27, and HSC4 cells using antibodies against the indicated proteins. GAPDH was used as the loading control **(C)** Colony formation assay displaying Cal27 and Tca8113 cells treated with DMSO or Fisetin for 14 d stably after being infected with lentivirus CON or Lv-MUC1. The number of cell colonies was counted and statistical analysis. **(D)** The scratch assay was used to detect the healing of 24 h in HSC4 cells. **(E)** The invasion ability was determined after Con or Lv-MUC1 in HSC4 cells treated with or without Fistein (40 μM) by using the Transwell assay. Left, representative images; right, quantification data.
